# Are Older People Really More Susceptible to SARS-CoV-2?

**DOI:** 10.14336/AD.2022.0130

**Published:** 2022-10-01

**Authors:** Shuo Zhang, Zhen Yang, Zhuo-Ning Li, Zhen-Lin Chen, Shi-Jun Yue, Rui-Jia Fu, Ding-Qiao Xu, Sai Zhang, Yu-Ping Tang

**Affiliations:** ^1^Key Laboratory of Shaanxi Administration of Traditional Chinese Medicine for TCM Compatibility, and State Key Laboratory of Research & Development of Characteristic Qin Medicine Resources (Cultivation), and Shaanxi Key Laboratory of Chinese Medicine Fundamentals and New Drugs Research, and Shaanxi Collaborative Innovation Center of Chinese Medicinal Resources Industrialization, Shaanxi University of Chinese Medicine, Xi’an, Shaanxi, China.; ^2^School of Clinical Medicine (Guang’anmen Hospital), Beijing University of Chinese Medicine, Beijing, China.; ^3^School of Public Health, Shaanxi University of Chinese Medicine, Xi’an, Shaanxi, China.; ^4^International Programs Office, Shaanxi University of Chinese Medicine, Xi’an, Shaanxi, China.

**Keywords:** SARS-CoV-2, COVID-19, older people, infection, age, ACE2, cytokine

## Abstract

Since the outbreak, COVID-19 has spread rapidly across the globe due to its high infectivity and lethality. Age appears to be one of the key factors influencing the status and progression of SARS-CoV-2 infection, as multiple reports indicated that the majority of COVID-19 infections and severe cases are elderly. Most people simply assume that the elderly are more susceptible to SARS-CoV-2 than the young, but the mechanism behind it is still open to question. The older and younger people are at similar risk of infection because their infection process is the same and they must be exposed to the virus first. However, whether they will get sick after exposure to the virus and how their disease progresses depend on their immune mechanisms. In older populations, inflammation and immune aging reduce their ability to resist SARS-CoV-2 infection. Meanwhile, under the influence of comorbidities, ACE2 receptor and various cytokines undergo corresponding changes, thus accelerating the entry, replication, and transmission of SARS-CoV-2 in the body, promoting disease progression, and leading to severe illness and even death. In addition, the relatively fragile mental state of the elderly can also affect their timely recovery from COVID-19. Therefore, once older people are infected with SARS-CoV-2, they are more prone to severe illness and death with a poor prognosis, and they should strengthen protection to avoid exposure to the virus.

COVID-19, a severe respiratory disease caused by SARS-CoV-2 infection [1], has been classified by the World Health Organization as a public health emergency of international concern because of its high infectiveness and lethality [2]. Since its outbreak, COVID-19 has spread rapidly and enveloped most of the world [3] and has seriously affected people’s daily life and work due to its strong infectivity and widespread. Multiple reports have shown that the death rate from COVID-19 increase exponentially with age [4], and the older people are at greater risk of contracting COVID-19 [5]. The majority of COVID-19 infected, and critically ill patients are elderly [6], and about 50% are over 60 years old [7]. Aging makes people more susceptible to chronic diseases and microbial infections [8-9]. Therefore, many people believe that the elderly are more susceptible to SARS-CoV-2 infections than the younger due to age, which seems to be one of the important factors affecting SARS-CoV-2 infection [10]. But why aging appears to have such a direct and clear relationship with COVID-19 is one of the greatest mysteries in medical field. The close link between advanced age and a higher risk of severe illness from COVID-19 is one of the most compelling information [11-12]. Based on the current data, it is true that the elderly account for a large proportion of the severe and death cases among COVID-19 patients, but are the elderly more susceptible to COVID-19 than the younger?

**Figure 1.**
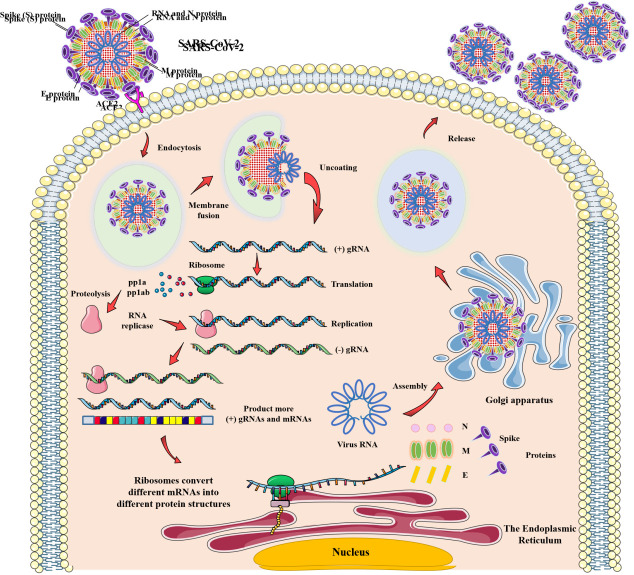
The process of SARS-CoV-2 entering human cells for replication and release.

## Older and younger people are at similar risk of SARS-CoV-2 infection based on the infection process

The main source of infection for COVID-19 is COVID-19 patients, who may be highly contagious before they show symptoms [13]. SARS-CoV-2 enters the respiratory tract after contacting the source of infection, and its S protein binds to angiotensin converting enzyme 2 (ACE2) in the epithelial cells of the respiratory tract to invade the body, then the virus enters the alveoli and infects alveolar epithelial type 2 (AET2) to help itself multiply and spread. After endocytosis, SARS-CoV-2 enters host cell, where it uncoats and releases (+) gRNA to initiate viral replication and inhibit the original gene expression of human cells. Ribosome translates (+) gRNA to form pp1a and pp1ab, which are hydrolyzed into RNA replicases. Then a large number of (+) gRNAs and mRNAs are produced under the action of RNA replicase, and ribosome translates different mRNAs to produce different viral proteins, which combine with (+) gRNAs to continuously form progeny SARS-CoV-2. More and more progeny viruses are produced, and they leave the host cell via golgi apparatus to attack other healthy cells and continue to infect and replicate. (Fig. 1) [14]. At this point, the virus is recognized by macrophages or dendritic cells in the alveoli and releases cytokines to present the antigen to T cells or other adaptive immune cells. The T cells, along with their corresponding receptors or other lymphocytes, then kill the infected cells to prevent the spread of the virus, and neutrophils migrate to the site of infection to remove the infected cell debris [15]. In addition to AET2 cells, ACE2 is also highly expressed in epithelial cells of the esophagus, ileum and colon, so the digestive tract may be a potential route for SARS-CoV-2 infection [16]. Similarly, because ACE2 is highly expressed in renal tubule cells, leydig cells and spermatogenic tubules, the virus may cause renal and testicular tissue damage [17].

**Figure 2.**
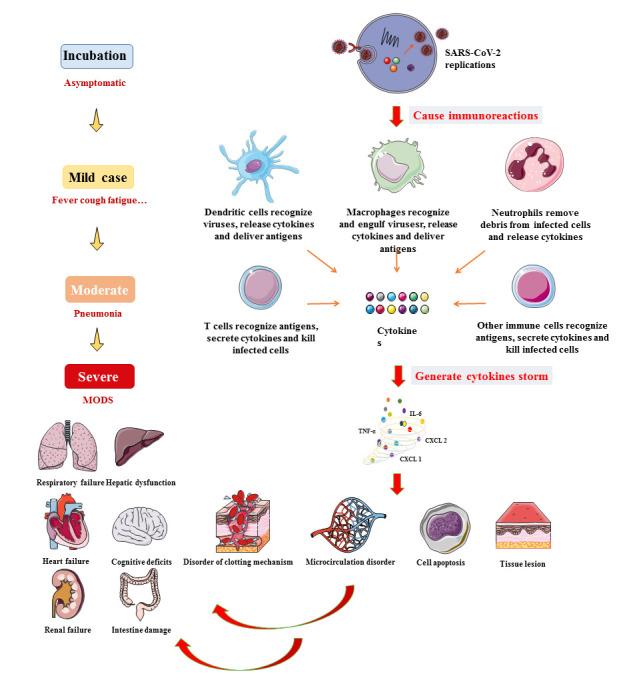
The severe disease pathogenesis caused by SARS-CoV-2 infection.

Under normal circumstances, the human immune system will respond quickly to the invasion of the virus, producing a large number of specific antibodies to resist virus replication. People with strong immune systems may have no symptoms or only mild tissue damage, called as asymptomatic infected persons. However, most of the infected patients have mild symptoms with a good prognosis, who are partially cured of mild pneumonia and can recover within a week [18]. In the initial stage, if the immune system does not respond in time or cannot resist the SARS-CoV-2, the virus will continue to replicate. With the massive reproduction of the virus and the infinite expansion of infection, multiple organs of the human body will gradually be involved and can cause multi-system symptoms, even life-threatening (Fig. 2). According to the mechanism of SARS-CoV-2 invasion in human body, it can be seen that all populations are susceptible to SARS-CoV-2 and the risk of infection is related to the risk of exposure. The process and links of pathogenesis are the same for all people, including virus exposure, entry into the body, replication and a series of symptoms and disease, but the incidence and severity of the disease are related to the body’s immune function. Therefore, young and old people can only become infected when exposed to the virus, and both of them are at a similar risk of infection.

**Figure 3.**
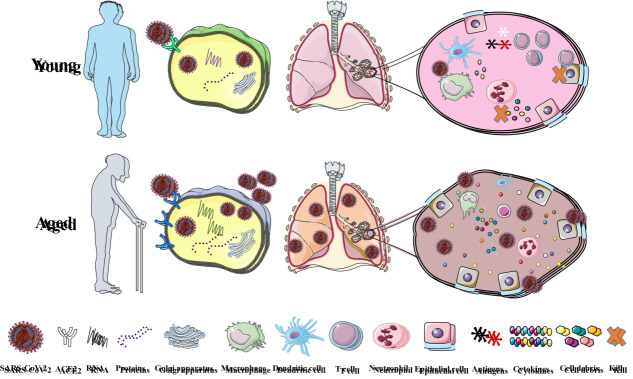
The different SARS-CoV-2 infection results for older and younger people.

## Older people infected with SARS-CoV-2 are more likely to develop disease

SARS-CoV-2 infection is contagious. It is unlikely to be infected if appropriate precautions are taken, but the outcome will be different for older and younger people once infected with the virus if not taken properly. For young people with strong immune function, although the virus invades the human body, the body can rapidly inhibit its replication, kill the infected cells, block the further development of the disease, and even heal itself, so it is not easy to be detected. Even if the disease occurs, it is mainly mild. However, in older people’s immune systems, the initial alarm signals are slowly released when the virus attacks the body, leading to more viral replication, so the virus will enter into the human body faster and more cells become infected. In addition, macrophages and T cells work less efficiently due to a lack of immune cells in older adults, which also leads to more cells being infected with viruses, resulting in high levels of inflammatory cytokine signaling. Therefore, the elderly are more prone to disease and aggravation after being infected with SARS-CoV-2. The most susceptible organ is the lung, where cytokine storms trigger microvascular coagulation, resulting in reduced lung volume, severe hypoxia, coagulation disorders and ARDS. If the treatment is not timely, SARS-CoV-2 will enter the bloodstream and further affect the whole body, causing heart failure, hepatic and kidney dysfunction, cognitive deficits and so on, which leads to multiple organ failure and even death with a poor prognosis (Fig. 3) [19].

SARS-CoV-2 binds to ACE2 enzymes on the epithelial cells of the upper respiratory tract, where they are absorbed and replicated internally, alerting the immune system, and the virus then enters the alveoli and infects type 2 lung cells. In the young system, macrophages or dendritic cells recognize viruses and provide antigens to T and other adaptive immune cells. T cells, together with appropriate receptors, activate other lymphocytes or kill infected cells directly to prevent the spread of the virus, and neutrophils migrate to the site of infection to remove infected cell debris. In aging systems, virus alarm signals are initially slow, leading to greater virus replication. Poor immune cell function prevents timely recognition and clearance of SARS-CoV-2, resulting in more cells to become infected and triggering high levels of inflammatory cytokine signaling, further exacerbating disease progression.

**Table 1:** Changes affecting the body’s immune system with age.

Immune system categories	Cell types	Affecting with age
**Innate immune system**	Macrophages	The ability to activate T cells (-)
		Oxidative burst (-)
		Release cytokines and chemokines (+)
	Dendritic cells	Pathogen recognition ability (-)
		Stimulate antigen specific T cells (-)
		Lymph node homing (-)
		Release cytokines and chemokines (+)
	Neutrophils	Responses to Stimuli (-)
		Phagocytic function (-)
		Respiratory burst generation (-)
		Disruptive oxidation (-)
		Bactericidal activity (-)
		Release cytokines and chemokines (+)
	NK cells	Immunologic function (-)
		Cytotoxicity (-)
		Release cytokines and chemokines (+)
**Adaptive immune system**	Lymphocytes	Production (-)
		Release cytokines and chemokines (+)
	T cells	The number, especially active T cells and costimulatory molecules (CD8, CD27, CD28, CD40L, etc.) (-)
		Diversity (-)
		Proliferative ability (-)
		Apoptosis resistance of memory cells (+)
		Release cytokines and chemokines (+)
	B cells	Production of cell precursors (-)
		The number, especially active T cells and costimulatory molecules (CD27, CD40, etc.) (-)
		Antibody affinity maturation (-)
		Isotype switch (-)
		Apoptosis resistance of memory cells (+)
		Release cytokines and chemokines (+)
	Antibodies	Production (-)
		Affinity (-)
		Adjustment ability (-)
		The response to a new antigen (-)

(-): decrease; (+): increase.

An important reason for SARS-CoV-2 transmission in humans is the lack of human immunity to it. People with normal immunity can quickly mobilize the body’s own immune function to resist the virus and prevent disease in a short time, but in the elderly population, inflammatory and immune aging make them more vulnerable to SARS-CoV-2 [20]. This aging may involve functional and structural changes in the immune system, manifested as a reduced ability to fight infection [21], and immune aging and inflammatory factors are associated with a series of age-related diseases [22]. Therefore, the pathogenesis of SARS-CoV-2 infection in the elderly may be related to the aging of immune function. It is shown that the neuro-endocrine-immune system maintains homeostasis and keeps the body healthy [23], but the function of the immune active cells of human body will gradually decline with age [24]. The body normally removes the damaged cells, but this process becomes slower and less efficient with age, and the accumulation of large numbers of senescent cells can weaken the body’s defenses, making it harder to fight off infected cells. When many factors of aging come together, they may destroy the immune system by the combined effects. Older people cannot develop a strong adaptive response in the presence of large numbers of SARS-CoV-2 strains that are extremely infectious and virulent, making them particularly vulnerable to breakthrough infections. As a result of the degeneration of immune function, the respiratory tract cilia movement ability of older people is reduced, and the function of defense and immune cell is decreased, then the body immune system function is declined, namely immune aging [25], which makes the imbalance of immunity in elderly infections more obvious. So, both innate and acquired immunity are irreversibly degraded, leading to a decrease in the body’s ability to resist SARS-CoV-2 (Table 1) [26]. Due to immune aging, harmful circulation in the body of the elderly is not well controlled, coupled with SARS-CoV-2 infection. Furthermore, harmful substances will not only destroy immune cells, but also other cells, thus accelerating the progress of the disease and causing damage to organs and tissues [27]. In addition to immune aging, the ageing of normal tissue structure in the human body is also associated with a higher incidence of COVID-19 in older persons. For example, changes in lung anatomical structure and muscle atrophy of the elderly will lead to changes in the physiological function of the respiratory system as they age. Reduced respiratory cilia movement ability, airway clearance ability and lung reserves will decrease the defense barrier function and make people more susceptible to illness after SARS-CoV-2 infection. Similarly, young people with weakened immune systems infected with SARS-CoV-2 are also susceptible to COVID-19, making them more prone to develop the disease than healthy people. Moreover, most elderly patients suffer from one or more comorbidities at the same time, which also reduces the body’s resistance to SARS-CoV-2 infection through different mechanisms of action and weakens the response ability to SARS-CoV-2, leading to multi-system symptoms [28].

ACE2 is the main determinant of SARS-CoV-2 entry and transmission [15]. The number and expression of ACE2 positive cells are also increased in each cell with age [29]. Furthermore, older people have a high proportion of hypertension, diabetes and other cardiovascular and cerebrovascular diseases. These patients use angiotensin converting enzyme inhibitors (ACEI) and angiotensin receptor blockers (ARB), which can up-regulate ACE2 receptor and increase the likelihood of morbidity after SARS-CoV-2 infection. Interleukin 7 (IL7) is one of the important components in SARS-CoV-2 cytokine storms. It is shown that IL7, as a marker of aging, is elevated in the heart, lung and vascular tissues of older individuals, which can induce the increase of ACE2 expression in vascular endothelial cells in a NF-κB dependent manner [30]. These findings provide a scientific basis for the increased incidence of COVID-19 associated with aging. At the same time, the cells of the regulatory system of the elderly show the greatest oxidative damage, as aging is a chronic oxidative stress process, thus it is unable to maintain their redox balance, resulting in the loss of function and inability to completely maintain the homeostasis in the body, which directly increases the probability of disease in the elderly [31]. Due to the degradation of various physiological functions, the disease resistance of older people is not as good as young, and the body’s defense function declines significantly. Therefore, when the older people are infected with SARS-CoV-2, the virus replicates and spreads faster, and it is more likely to produce symptoms and accelerate the progression of the disease. They are at high risk of illness after being infected with SARS-CoV-2 [32].

## Older people with COVID-19 are more likely to become severe

The main symptoms of COVID-19 are fever, cough, pneumonia and myalgia, and the respiratory and cardiovascular systems are its most vulnerable tissues, causing tachypnea, hypoxemia and coagulopathy [33], which can be significantly aggravated in elderly patients [34]. Studies have found that advanced age is an independent risk factor for COVID-19 mortality and severe infection [35], and elderly patients with comorbid diseases account for a large proportion of severe and fatal patients [36]. Approximately 50% of hospitalized COVID-19 patients have pre-existing conditions, including diabetes, hypertension, obesity and metabolic syndrome, cardiovascular disease and chronic obstructive pulmonary disease, which are closely associated with greater disease severity and mortality after SARS-CoV-2 infection [18-19,37]. For examples, hyperglycemia can increase the plasma osmotic pressure, as well as inhibit the chemotactic activity, phagocytosis and intracellular killing of leukocytes, thereby reducing the body’s resistance to infection and weakening the reaction ability to the invasion of viruses [38]. And long-term chronic airway diseases can cause squamous metaplasia of ciliated columnar epithelium of airway mucosa, increased mucus glands and decreased mucosal barrier defense function. Thus, it is easy to develop into severe disease once infected [39]. And patients with severe COVID-19 are at higher risk of respiratory and cardiovascular problems. SARS-CoV-2 infection in older people can lead to multiple serious and fatal complications [40] or worsen existing diseases, which is the leading cause of death. The mean age of severe COVID-19 patients is over 70 [36], and the case fatality rate of patients over 60 is 4.5%, which is significantly higher than that of patients under 60 (1.4%). Moreover, patients with comorbidities account for 67% of deaths, including 48% of hypertension, 31% of diabetes and 13% of coronary heart disease, and people with two or more comorbid diseases are more likely to develop severe diseases [41].

**Figure 4.**
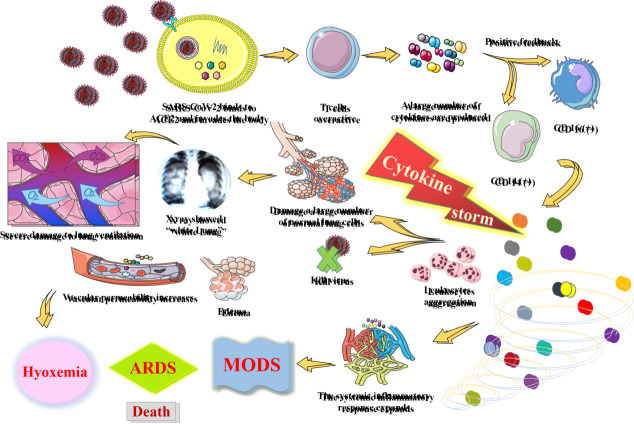
Cytokine storm mechanism induced by SARS-CoV-2.

The severity of COVID-19 is associated with immune system dysfunction in older people, and recently, immune aging has been identified as a key factor in exacerbating COVID-19 [42], and cytokine storm is also associated with COVID-19 severity [18], which plays a very important role in the progression of COVID-19 [43]. The lethality of COVID-19 depends on systemic hyperinflammation caused by cytokine storms [44]. When viruses and cytokines enter into the bloodstream, cytokine storms can trigger microvascular clotting and lead to severe oxygen deprivation in the lungs, clotting disorders and organ failure [45]. When the virus replicates in large numbers, hyperactive monocytes and macrophages infiltrate the lungs, causing oversecretion of these cytokines, which results in a rapid and intense cytokine storm that leads to white blood cell aggregation, vascular permeability reduction, edema and further damage to other organs and ultimately death (Fig. 4). Among them, neutrophil hyperplasia, coagulation activation and acute kidney injury may be closely related to the death of COVID-19 patients [46].

Chronic inflammation caused by mitochondrial dysfunction is responsible for the explosive release of inflammatory factors in COVID-19 patients, leading to severe pneumonia, multiple organ failure and eventual death, and it is the key factor in the severity of COVID-19 [47]. Mitochondrial dysfunction is closely related to aging and is the main progression of aging and age-related diseases, which plays a very important role in a variety of comorbid diseases [48]. Improved mitochondrial activity in older people can be used as a preventive therapy to improve immune response and reduce mortality from respiratory diseases [49]. COVID-19 is also associated with high levels of interleukin-6 (IL-6) and other pro-inflammatory cytokines [50-51]. Chitinase 3-like-1 (CH3L1) is a risk factor for severe COVID-19 and is increased significantly during infection. Once infected with SARS-CoV-2, the progression of the disease will be quickly accelerated. It is shown that CH3L1 level increases with age and comorbidities, and it is the best predictor of all-cause mortality in people over 80. Moreover, CH3L1 is an effective SARS-CoV-2 stimulator that acts on human body through ACE2 receptor, and this induction is the main mechanism by which aging aggravates the COVID-19 disease [52]. CH3L1 levels are significantly elevated in elderly patients with COVID-19 or comorbidities and in severe COVID-19 patients, expressed in exaggerated ways as a risk factor for COVID-19 in aging and comorbidities [53], so CH3L1 provides a key explanation for how aging, and comorbidities aggravate the progression of COVID-19. ACE2 expression levels also play a key role in determining the extent of infection and organ location, as well as the severity of the disease [54], and the shared oxidative stress is also associated with increased overall mortality [55]. Therefore, immune aging and a number of comorbidities in the elderly are the key to the deterioration of the immune response to SARS-CoV-2 infection, which prevents successful suppression of viral transmission in the early stages of infection and thus increases severe morbidity and mortality in this population.

## The relatively fragile psychological state also influences the timely recovery of the older COVID-19 patients

COVID-19 has a devastating effect on older people [56]. The proportion of death and severe illness among elderly patients has been extremely high. Although a large part of them have been fully vaccinated against COVID-19, they are still the most high-risk group for the disease and critical illness because their immune antibodies decline or even disappear quickly. It is shown that the efficacy, immunogenicity and antibody duration of vaccines in the elderly are lower than those of in the young [57-58]. Furthermore, immunization programs that work well in healthy populations may not be appropriate or adequate for the elderly because most vaccines evaluated in early clinical trials were conducted in healthy young individuals. In addition, most elderly people suffer from comorbid diseases, which are generally considered as contraindication for vaccination. Thus, the use of vaccines in the elderly still requires special consideration, and the prevention and control of COVID-19 in the elderly should focus on avoiding exposure to the virus. After the elderly are infected with SARS-CoV-2, they are prone to multiple symptoms and aggravation of the disease due to the complexity of various diseases and combined effects of psychosomatic factors. Furthermore, elderly COVID-19 patients are also difficult to cure, and even suffer from a long and protracted course of disease, as well as repeated exacerbation. It is shown that individuals’ awareness of the epidemic can affect their mental health status and epidemic prevention behavior, and the public’s health awareness of epidemic information and prevention methods can affect their psychological status [59]. Older people are experiencing more psychological stress and health challenges during the COVID-19 pandemic due to their increased susceptibility to severe cases of SARS-CoV-2 infection and strict isolation requirements imposed during the pandemic [60]. With the addition of age, physiological aging is inevitably accompanied by psychological aging. As a vulnerable group, the elderly are exhausted physically and mentally, and their psychological change limits the living ability, activity ability and strain capacity, which reduces the independence of life, affects the life quality and makes their mentality extremely fragile and sensitive. In particular, the elderly are gradually isolated from the society after leaving their jobs. They are less social and have few people to talk to, which prevents many of their emotions from venting in time. The number of empty nesters is increasing, and older people often feel lonely and spiritually empty [61]. In the face of COVID-19, a new type of sudden and catastrophic public event, the elderly are even more confused and their anxiety about SARS-CoV-2 infection is related to more stress caused by COVID-19 compared with younger people [62], which may cause changes in the body to some extent, thus aggravating comorbid diseases or further weakening immunity. When infected with SARS-CoV-2, older people are more likely to become ill, accelerating the disease progression and developing severe cases. At the same time, under the influence of many factors that includes lack of medical knowledge, physical pain caused by disease, isolation from loved ones, changes in the surrounding environment and repeated medical intervention and monitoring, the elderly are more prone to have negative emotions such as fear, anxiety, depression, resulting in post-traumatic stress disorder [63], which is not conducive to the recovery of the disease and may aggravate the illness and prolong the course of the disease.

Older people are most affected by COVID-19, especially those with comorbid disease. With the addition of age, the physiological function of the elderly naturally ages and gradually degrades. Firstly, the immune function of older people is decreased, leading to the reduction of resistance to external sources of disease. Once older people are exposed to the virus, it will quickly act on the human body with high frequency of diseases. Secondly, the ability of self-regulate rehabilitation is weakened if the disease occurs, and the body function of older people is difficult to restore to the original state, so the rate of cure and recovery is low. Thirdly, there are many comorbid diseases. Their metabolic capacity declines due to the older age, and various complications often occur. In addition, the virus can be life-threatening in the elderly. SARS-CoV-2 is generally susceptible to all groups of people, and the risk of infection is similar between older and younger adults. The susceptible organs and infection routes of the elderly and the young are roughly the same. Most viruses enter the human body from the epithelial cells of the respiratory tract and infect the lungs first, then further other organs through the blood circulation. But symptoms differ between the elderly and the young COVID-19 patients. Fever, cough, fatigue, headache, sore throat, myalgia, nausea, diarrhea, and loss of taste and smell are the most common symptoms of COVID-19 patients. Young people who are infected with COVID-19 have few or mild symptoms, which can be shown as low fever or even no fever with only individual symptoms or even no obvious symptoms, while the elderly may have numerous and more severe symptoms, manifested as persistent high fever, severe cough, fatigue and symptoms of multiple organs. So, it is clear that the elderly are most likely to have complex symptoms and are at greatest risk of death. After exposure to SARS-CoV-2, the older tend to get sick and become severe. In the elderly, the disease is affected by low immunity, more comorbid diseases and great psychological stress, which progresses quickly with a long course of disease and a high fatality rate and is prone to recurrent attacks and extremely difficult to recover. Therefore, in the COVID-19 pandemic, the older people are experiencing higher morbidity, severe illness and fatality rates than the young [64], and they should be the focus of the prevention and treatment of COVID-19. However, their current literacy level is lower than that of the population as a whole. More importantly, in terms of health issues, the prevention and control of infectious disease among the elderly is at the lowest level of literacy. As an infectious disease, COVID-19 is one of the blind spots in the health knowledge of the elderly, and their awareness of the dangers and infectivity of COVID-19, as well as their behavior in taking relevant preventive measures, may lag behind the younger population. Studies have found that compared with younger people, the elderly are more likely to agree that people are overreacting to the threat of COVID-19, and they believe it’s really no different from flu [65]. Therefore, during the epidemic period, attention should be firstly paid to improving the awareness of epidemic prevention ideology of the elderly, and their related knowledge and psychological response should not be ignored.

As there is currently no cure or prevention method for COVID-19, in addition to vaccination, the main means of epidemic prevention is to change daily behaviors, and the health policy of combining prevention and treatment should still be adhered to [46]. Wearing masks, maintaining social distance and isolation are still the key to epidemic prevention and control [66]. Moreover, the characteristics of multi-system diseases in the elderly lead to the complexity of the disease and mutual influence. Once COVID-19 is diagnosis, the difficulty of treatment will be greatly increased. At present, SARS-CoV-2 has emerged in a variety of variants. The Alpha, Beta, Delta, Gamma and Omicron have been reported by WHO as strains of concern, and the Epsilon, Eta, Iota, Kappa and Zeta as variants of interest. These variants enhance the infectivity and infection rates of COVID-19, especially in the elderly, and the mutated virus may become resistant to existing vaccines, reducing their protection in the elderly. Therefore, in the context of regular epidemic prevention and control, compared with young people, the elderly should pay more attention to protection, strengthen awareness of epidemic prevention, avoid contact with the virus, and implement self-health management behaviors that highlight epidemic prevention concepts according to their comorbid diseases. In addition, maintaining good living habits and positive attitude, as well as reducing anxiety, depression and other episodes of the elderly are also conducive to the prevention and treatment of COVID-19. For the elderly without comorbidities, on the basis of protection, they can inject corresponding COVID-19 vaccine and timely get booster shots to maintain antibody levels and improve immune function. For patients with other comorbidities, it is recommended to take daily protection and frequent disinfection as the main measures for the prevention and control of COVID-19, and they also should actively treat comorbid diseases to improve physical condition. If there is a history of epidemiological exposure, older people should be highly vigilant. As long as there are suspected symptoms such as fever, cough, dyspnea, poor appetite or even changes in consciousness, the nearest fever clinic should be consulted, and the diagnose should be actively performed through imaging and molecular biology examination [67]. This is the only way to better protect the older people from COVID-19.
